# Contextual dimensions of pediatric tuberculosis imaging: radiation exposure, access, and system capacity in high- and low-resource settings

**DOI:** 10.1007/s00247-026-06535-z

**Published:** 2026-02-18

**Authors:** Isabelle Munyangaju, Andreas Jahnen, Ridwaan Esmail, Benedita José, Jacinta Adrigwe, Criménia Mutemba, Patricia Pérez, José Miguel Escudero Fernández, Antoni Soriano-Arandes, Maria Espiau, Begoña Santiago Garcia, Alicia Hernanz-Lobo, Ángel Lancharro-Zapata, Aleix Soler-Garcia, Enrique Ladera, Antoni Noguera-Julian, Angela Manzanares, Daniel Blazquez, Elisa Aguirre Pascual, Quique Bassat, Elisa Lopez-Varela, Isabelle Thierry-Chef

**Affiliations:** 1 Barcelona Institute for Global Health, C. Rosello 171, 1, 08036 Barcelona, Spain; 2 Facultat de Medicina i Ciències de la Salut, University of Barcelona, Barcelona, Spain; 3 Luxembourg Institute of Science and Technology, Belvaux, Luxembourg; 4 Radiation Oncology, Ministry of Health, Maputo, Mozambique; 5 National Tuberculosis Control Program, Ministry of Health, Maputo, Mozambique; 6 National Paediatric TB Working Group, Maputo, Mozambique; 7 Tinpswalo Association: Vincentia Association to Fight AIDS and TB, Maputo, Mozambique; 8 Radiologia Pediàtrica, Vall d’Hebron Hospital Universitari, Barcelona, Spain; 9 Unitat de Patologia Infecciosa i Immunodeficiències de Pediatria (UPIIP), Vall d’Hebron Hospital Universitari, Barcelona, Spain; 10 Infección e Inmunidad en el paciente pediátrico, Vall d’Hebron Institut de Recerca, Barcelona, Spain; 11 IiSGM - Instituto de Investigación Sanitaria Gregorio Marañón, Hospital General Universitario Gregorio Marañón, Madrid, Spain; 12 Centro de Investigación Biomédica en Red de Enfermedades Infecciosas (CIBERINFEC), Instituto de Salud Carlos III, Madrid, Spain; 13 Radiologia Pediàtrica, Hospital General Universitario Gregorio Marañón, Madrid, Spain; 14 Malalties Infeccioses i Resposta Inflamatòria Sistèmica en Pediatria, Servei de Malalties Infeccioses i Patologia Importada, Institut de Recerca Pediàtrica Sant Joan de Déu, Barcelona, Spain; 15 Radiologia Pediàtrica, Hospital Sant Joan de Déu Barcelona, Barcelona, Spain; 16 Red de Investigación Transalacional en Infectología Pediátrica (RITIP), Madrid, Spain; 17 Unidad de Enfermedades Infecciosas Pediátricas. Hospital Universitario 12 de Octubre. Instituto de Investigación Hospital 12 de Octubre. Universidad Complutense, Madrid, España, Madrid, Spain; 18 Radiologia Pediàtrica, Hospital Universitario 12 de Octubre, Madrid, Spain; 19 Institució Catalana de Recerca i Estudis Avançats (ICREA), Barcelona, Spain; 20 Autorité de Sûreté Nucléaire et de Radioprotection, 17, 92262 Fontenay-Aux-Roses cedex, Paris, France

**Keywords:** Pediatric tuberculosis, Chest radiography, Computed tomography, Radiation dose, Imaging access, Low-resource settings

## Abstract

**Background:**

Pediatric tuberculosis diagnosis relies heavily on imaging, yet access, equipment standards, and dose monitoring differ widely across health systems. Evidence describing how these contextual factors influence imaging use and radiation exposure in children remains scarce.

**Objective:**

To describe pediatric tuberculosis imaging practices and estimated radiation doses across two distinct resource settings, Spain (hospital-based, high-resource) and Mozambique (primary care-based, low-resource), to inform strategies for safe, equitable, and context-appropriate imaging.

**Methods and materials:**

A descriptive mixed-methods study combined retrospective data of children (<16 years) diagnosed with tuberculosis (Spain 2015–2021; Mozambique 2018–2021) with complementary surveys of imaging providers. In Spain, chest X-ray and computed tomography parameters were extracted from digital imaging and communications in medicine files to estimate organ-specific doses using the National Cancer Institute dosimetry systems for radiography and computed tomography. In Mozambique, dose estimates were based on standardized pediatric protocols and site survey parameters due to limited digital data. Surveys captured information on imaging access, guideline use, and professional training.

**Results:**

Imaging data were available for 84 Spanish and 83 Mozambican children. In Spain, children underwent multiple chest X-rays (mean four per child) and computed tomographies (mean three per child), resulting in cumulative lung doses up to ~20 mGy cm^2^, remaining below diagnostic reference levels. In Mozambique, most children had one or two chest X-rays, with cumulative lung doses <0.05 mGy cm^2^. Survey findings indicated structured dose optimization and quality assurance practices in Spain, versus limited equipment and predominantly non-physician interpretation in Mozambique.

**Conclusion:**

Context-appropriate improvements in pediatric imaging such as strengthened infrastructure, training, dose monitoring, and quality assurance are essential to ensure safe exposure and equitable, reliable tuberculosis diagnosis for children.

**Graphical abstract:**

**Figure:**
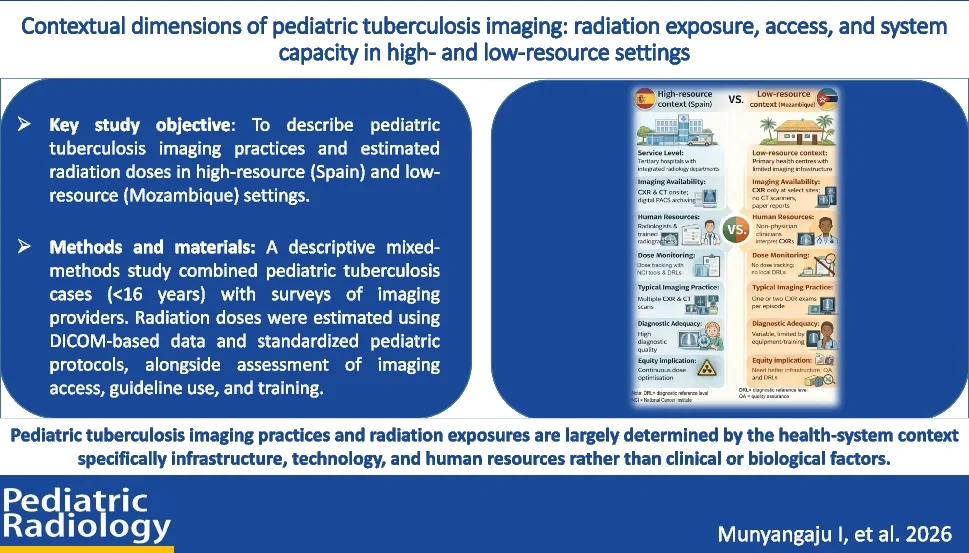

**Supplementary Information:**

The online version contains supplementary material available at https://doi.org/10.1007/s00247-026-06535-z.

## Introduction

Tuberculosis (TB) remains a major global health concern, including among children, where it continues to cause significant morbidity and mortality despite being preventable and treatable [1]. Diagnosing TB in children is particularly challenging because clinical manifestations are often nonspecific, microbiological confirmation is frequently difficult, and diagnostic algorithms rely heavily on clinical and radiological findings [2, 3]. Recent pediatric TB imaging guidelines further emphasize structured imaging pathways and standardized algorithms for thoracic TB evaluation, highlighting the central role of chest radiography (chest X-ray (CXR)) and appropriate use of adjunct modalities [4–6].

Radiological imaging provides critical information to support early diagnosis and guide treatment, but repeated exposures in children raise important considerations regarding radiation protection. Children are more radiosensitive than adults and have a longer lifetime in which potential radiation-induced effects can manifest [7, 8]. Pediatric organs such as the thyroid, breast, skin, and bone marrow are particularly sensitive to ionizing radiation, underscoring the need for adherence to international dose optimisation principles [7].

The World Health Organization (WHO) recognizes CXR as a key diagnostic tool for TB because it is widely available, low-cost, and highly informative when interpreted by trained clinicians [9]. Typical pediatric CXR doses are low (0.01–0.1 mGy cm^2^), depending on equipment and patient size [10, 11]. However, advanced imaging modalities such as computed tomography (CT) can deliver much higher organ doses (ranging from <0.1 mGy cm^2^ to >50 mGy cm^2^) depending on scan type and protocol [12, 13]. While CT provides detailed anatomical information and can detect complex or extrapulmonary manifestations [14], its use in children must follow strict justification criteria consistent with the “as low as reasonably achievable” (ALARA) principle [15, 16].

Imaging practices vary between high-income countries, where both CXRs and CT scans are routinely used for TB diagnosis and follow-up [5, 14], and low-income settings, where CXRs are the primary imaging tool due to resource constraints [17]. To date, few studies have systematically described how pediatric TB imaging is conducted in diverse resource settings or documented the contextual differences in estimated radiation exposure across them [14, 18–20]. Understanding such variations is essential for tailoring radiation protection measures, developing locally appropriate diagnostic reference levels, and strengthening the quality of TB imaging in children.

This study aimed to describe and contrast pediatric TB imaging practices, access, and estimated radiation doses across two distinct healthcare contexts: Spain, representing a high-resource hospital-based system, and Mozambique, representing a primary care-based low-resource system. This study examined whether and how variations in infrastructure, imaging protocols, and professional training between distinct healthcare contexts might shape pediatric TB imaging use and associated radiation exposure. Understanding these contextual influences was expected to provide a foundation for developing tailored strategies to optimize radiation dose, improve image quality, and strengthen diagnostic equity in pediatric TB care.

## Methods

This descriptive mixed-methods study assessed pediatric TB imaging practices and estimated cumulative radiation doses CXR and CT (only in Spain) in two distinct healthcare contexts: Spain (a high-resource, hospital-based system) and Mozambique (a low-resource, primary care-based system) [21]. The study design sought to document contextual differences in imaging frequency, protocols, and estimated dose exposure rather than to perform a direct clinical comparison.

In Spain, data were obtained from four tertiary hospitals (two in Madrid and two in Barcelona) participating in the pediatric TB research network (pTBred) [22]. Although these hospitals are tertiary referral centers, they reflect routine pediatric TB service delivery in Spain, where most children with suspected or confirmed TB are evaluated in specialized hospital-based settings. In Mozambique, five primary health centers (two in Maputo City and three in Maputo Province) were selected to represent routine pediatric TB service delivery in low-resource primary care settings. These sites reflect contrasting service levels where pediatric TB diagnosis is mainly hospital-based in Spain and primary care-based in Mozambique.

Two complementary surveys were conducted to capture contextual information on imaging protocols and clinical use:

- A site-level survey of radiologists (medical doctors specialized in radiology) and radiographers (medical imaging technologists) collected technical details on standard pediatric CXR protocols. These data were used to estimate radiation doses where patient-level digital imaging and communications in medicine (DICOM) information was unavailable, particularly in Mozambique.
- An anonymous provider survey targeted healthcare professionals involved in pediatric TB care in both countries (Mozambique, *n*=68; Spain, *n*=36) to document access to imaging, guideline use, and professional background. Respondents were recruited through institutional networks and snowball sampling. Both surveys were voluntary, anonymous, and followed ethical standards (Supplementary Material 1).

Data were collected in two phases. Phase 1 retrieved clinical data retrospectively for children younger than 16 years diagnosed with TB (pulmonary or extrapulmonary) during 2015–2021 in Spain and 2018–2021 in Mozambique. In Spain, cases were extracted from the multicentre pTBred database. In Mozambique, cases were identified from TB registers and clinical files, cross-checked against imaging logs when available. Variables included demographics, HIV status, TB form (pulmonary or extrapulmonary), microbiological confirmation, drug resistance, and imaging performed during diagnosis or follow-up. Cases without confirmed TB after data triangulation were excluded. Missing values were resolved through facility record review.

Phase 2 collected radiological parameters to estimate radiation dose. In Spain, *DICOMInspector* was used to extract technical metadata from hospital Picture Archiving and Communication System (PACS), enabling calculation of individual radiation doses per examination [23]. In Mozambique, without DICOM files or PACS, imaging parameters came from site surveys at the two X-ray-equipped facilities. Age-specific standard protocols and survey-reported CXR frequencies were used, so radiation estimates relied on standardized assumptions rather than patient-specific data.

### Dose estimation

Radiation doses were calculated using the National Cancer Institute (NCI) dosimetry tools *NCIRF Beta 3.0.20240315* for Radiography and Fluoroscope and *NCICT 3.0.20240626* for CT [24, 25]. To estimate organ radiation doses from pediatric chest imaging, the NCIRF and NCICT software required several key radiological input parameters.

For CXR dosimetry, essential input parameters included patient age and sex (which determine the anatomical phantom used), projection view (e.g., PA or AP), tube voltage (*kVp*), tube current-time product (*mAs*), focus-to-skin distance (*FSD*), source-to-image distance (*SID*), beam filtration, and the field size and location. The NCIRF software estimated organ doses using the dose area product (*DAP*, in *Gy* *cm*^*2*^) as the primary input, which represents the total absorbed dose multiplied by the irradiated area and is the standard unit for quantifying exposure in radiography. These parameters allowed individualized dose estimation, accounting for patient size and beam geometry. In Spain, most of these parameters were directly extracted from DICOM files, with missing values supplemented by site-specific, age-dependent, and kVp-dependent estimates from survey data. DICOMInspector was used to batch-process input files, which were then analyzed through NCIRF to compute organ-specific doses.

In Mozambique, radiation estimates relied on standardized assumptions from age-specific *kVp* and *mAs* values collected in site surveys. *Field size* and *FSD* were manually measured, and *X-ray tube output *(*mGy/mAs at a 1-m distance*) was provided by radiology departments. These inputs were used to calculate DAP as follows:

$$\text{Entrance skin dose }(\mathrm{ESD})=\mathrm{Output}\times \mathrm{mAs}\times {(100/\mathrm{FSD})}^{2}$$

$$\text{DAP }(\text{mGy }{\mathrm{cm}}^{2})=\mathrm{ESD}\times \text{field area }({\mathrm{cm}}^{2})$$

DAP values were then converted to Gy cm^2^ to align with NCIRF input requirements (supplementary material 2). Standard beam geometry for pediatric CXR was defined according to age-specific field sizes derived from DICOM data in Spain and manual measurements in Mozambique. Technique-related parameters such as projection (posterior-anterior/anterior-posterior), patient positioning, grid use, automatic exposure control, and collimation were not consistently available across sites and therefore could not be incorporated into dose estimation.

CT dosimetry required scan type, anatomical region, patient demographics, and scanner parameters: *Computed Tomography Dose Index volume* (*CTDIvol*, the average dose per slice) and *dose-length product *(*DLP*, the total scan dose). These values, obtained from DICOM metadata or scan logs in Spain, were processed in NCICT software to calculate patient-specific organ doses under pediatric imaging protocols.

Parameters were extracted from DICOM headers using DICOMInspector, and chest CT protocols were mapped by scanner type to define typical settings. Missing scan range data were inferred from protocol mapping and European Pediatric CT Radiation Dose Cohort (EPI-CT) results (a large multicountry study that provides standardized pediatric CT protocol and dose data) [26]. Organ doses were calculated in NCICT using weight- and height-specific phantoms with a uniform tube current modulation factor of 0.25. Missing *kVp* and/or *pitch* values were assigned from protocol mapping and EPI-CT data. When *CTDIvol* was available, batch files were processed in NCICT; otherwise, doses were estimated manually in NCICT using available imaging parameters. No CT scans were performed in Mozambique due to lack of equipment at the study sites; consequently, CT-related dose estimation was limited to the Spanish cohort.

Descriptive statistics were used to summarize patient characteristics, imaging frequency, and estimated radiation doses. Continuous variables were reported as mean, standard deviation, minimum, and maximum; categorical variables as proportions. Chi-square or Fisher’s exact tests assessed associations in demographic or clinical variables (*P*<0.05). No inferential statistical testing was applied to organ dose data because of small sample sizes and contextual heterogeneity between settings.

The study was approved by the ethics committees of all participating institutions in Spain and Mozambique (six in total; reference numbers available in acknowledgments). All approvals adhered to the Declaration of Helsinki and national regulations on research involving human participants. Patient data were anonymized before analysis. Both survey components were voluntary, and participation implied informed consent.

For the purposes of this analysis, participants were grouped into two cohorts following the World Bank income classifications [27]: a high-income country (high-resource context; HIC) cohort (Spain) and a low-income country (low-resource context; LIC) cohort (Mozambique) to emphasize how health-system context, rather than geography, shapes imaging access, dose estimation feasibility, and adherence to radiation safety principles.

## Results

Results are presented descriptively by setting, without formal statistical comparison, due to differences in data granularity, imaging availability, and different dose estimation methods.

### Overview of imaging practices and respondent characteristics

Survey responses were obtained from 36 providers in the HIC setting and 68 in the LIC setting. Participants in the HIC were all hospital-based physicians, mainly pediatricians and general practitioners, with 58% reporting 15–30 years of professional experience. In the LIC, most respondents (90%) worked in primary health centers, and 88% were non-physician clinicians such as medical assistants and nurses, 90% of whom had fewer than 15 years of experience.

Access to imaging differed substantially by context. In the HIC, all facilities had on-site CXR and CT, and imaging was interpreted by radiologists. In the LIC, only one-third of centers had on-site X-ray capability, and more than half reported transferring patients to other facilities for imaging. No CT scanners were available at the study sites. CXRs were mainly interpreted by non-radiologist clinicians (87%), and 81% of respondents reported the presence of local CXR guidelines, though standardization was variable (Supplementary Material 1 Tables 1–4).

Training and confidence in image interpretation also varied. In the HIC, nearly all clinicians had received formal CXR training through clinical rounds or radiology courses, whereas in the LIC, 37% had never received any structured training. Awareness of radiation safety was limited in both contexts but especially in the LIC, where 82% of respondents (vs. 50% of respondents in the HIC) could not estimate the approximate radiation dose of a pediatric CXR (Supplementary material 1 Tables 5–7).

### Characteristics of pediatric tuberculosis cases

A total of 84 children with TB and available imaging data were included from the HIC (2015-2021) and 83 from the LIC (2018-2021).

In the HIC, the mean age was 6.9 years (range 0.4–15.9.4.9), and 92% had pulmonary TB. Most were hospital referrals (83%), and 43% had bacteriological confirmation. In the LIC, the mean age was similar (6.9 years; range 0.3–15.3), but 89% presented with pulmonary TB and 17% were identified through community or primary-care screening. Laboratory testing was performed in 63% of cases, with 4% confirmed. HIV testing was universal in the LIC (42% positive), but incomplete in the HIC (63% not tested).

**Fig. 1:**
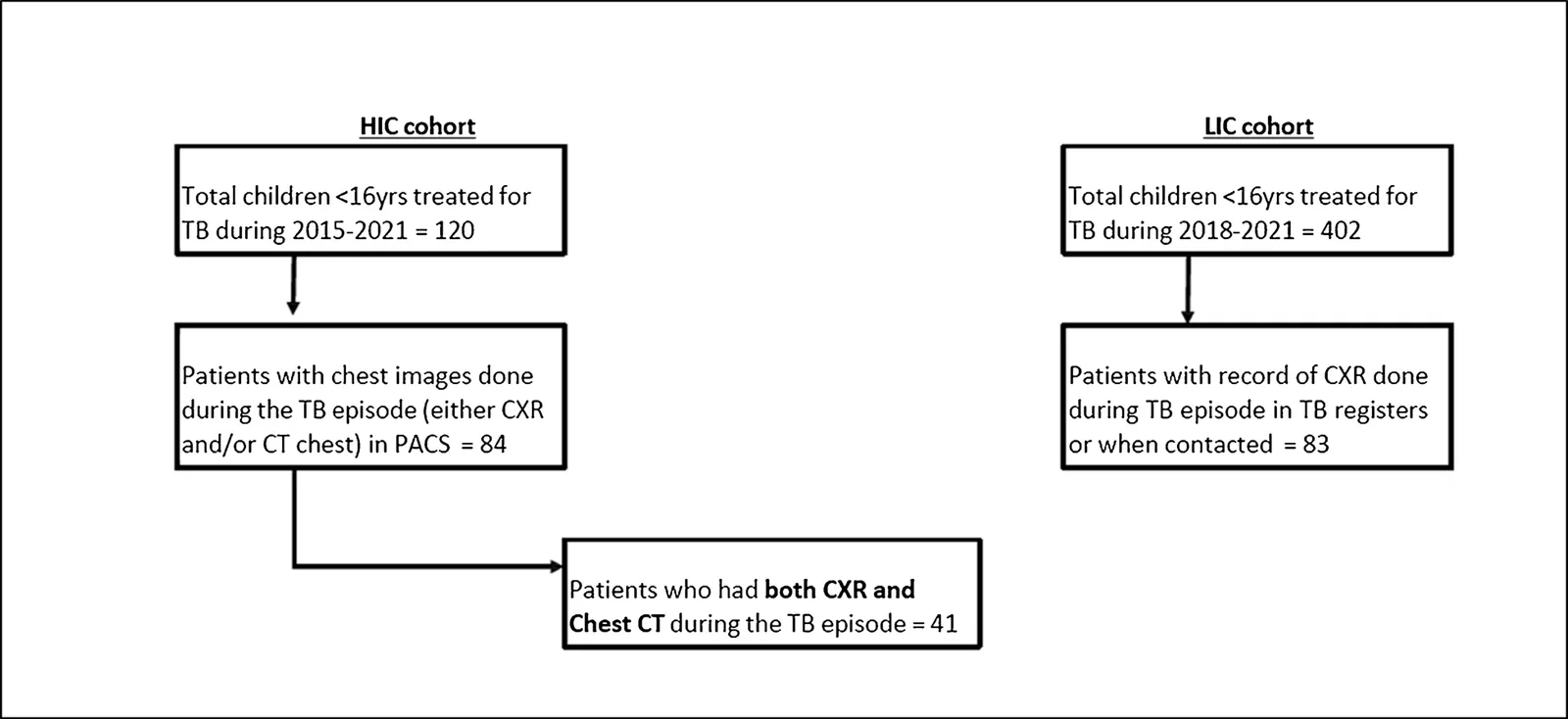
Flowchart of patient recruitment. Flow diagram summarizing the inclusion of pediatric tuberculosis cases with available imaging data from both study settings. Both datasets were independently assembled under distinct health-system conditions, reflecting contextual differences in record completeness and imaging documentation. *CT* computed tomography, *CXR* chest x-ray, *HIC* high-resource context (Spain), *LIC* low-resource context (Mozambique), *PACS* picture archiving and communication system, *TB* tuberculosis

Follow-up imaging was systematically documented in the HIC but not in the LIC, where the absence of archiving systems prevented longitudinal linkage (Table 1; Fig. 1).

**Table 1:** Clinical characteristics of pediatric tuberculosis patients: summarizes the demographic and clinical profiles of pediatric tuberculosis patients in each context

Characteristic	HIC total (*n*=84)	HIC female (*n*=55)	HIC male (*n*=29)	LIC total (*n*=83)	LIC female (*n*=33)	LIC male (*n*=50)	*P*-value (HIC/LIC)
**Age**							0.21^a^/0.32^a^
<1 yr	3 (4)	1 (2)	2 (7)	7 (8)	3 (9)	4 (8)	
1–4 yrs	31 (37)	24 (44)	7 (24)	25 (30)	7 (21)	18 (36)	
5–9 yrs	29 (35)	16 (29)	13 (45)	24 (29)	12 (36)	12 (24)	
10–14 yrs	17 (20)	12 (22)	5 (17)	21 (25)	7 (21)	14 (28)	
15+ yrs	4 (5)	2 (4)	2 (7)	6 (7)	4 (12)	2 (4)	
**Referral source**							0.36^a^/0.35
Hospital/other	70 (83)	44 (80)	26 (90)	69 (83)	29 (88)	40 (80)	
PHC/community	14 (17)	11 (20)	3 (10)	14 (17)	4 (12)	10 (20)	
**HIV status**							0.89/0.06
Negative	31 (37)	20 (36)	11 (38)	48 (58)	15 (45)	33 (66)	
Positive	–	–	–	35 (42)	18 (55)	17 (34)	
Not done	53 (63)	35 (64)	18 (62)	–	–	–	
On ART (if positive)	–	–	–	34 (97)	17 (94)	17 (100)	–/1.00^a^
**TB lesion**							0.69^a^/0.31^a^
Extrapulmonary	7 (8)	4 (7)	3 (10)	9 (11)	2 (6)	7 (14)	
Pulmonary	77 (92)	51 (93)	26 (90)	74 (89)	31 (94)	43 (86)	
**TB category**							0.69^a^/1.00^a^
New case	77 (92)	51 (93)	26 (90)	79 (95)	31 (94)	48 (96)	
Other	7 (8)	4 (7)	3 (10)	4 (5)	2 (6)	2 (4)	
**Resistance profile**							0.38^a^/0.56^a^
Resistant	7 (8)	3 (5)	4 (14)	3 (4)	2 (6)	1 (2)	
Sensitive	25 (30)	18 (33)	7 (24)	80 (96)	31 (94)	49 (98)	
Unknown	52 (62)	34 (62)	18 (62)	–	–	–	
**Lab test done**							0.27^a^/0.44
No	3 (4)	1 (2)	2 (7)	31 (37)	14 (42)	17 (34)	
Yes	81 (96)	54 (98)	27 (93)	52 (63)	19 (58)	33 (66)	
**Bacteriological confirmation**							0.75/1.00^a^
No	46 (57)	30 (56)	16 (59)	50 (96)	18 (95)	32 (97)	
Yes	35 (43)	24 (44)	11 (41)	2 (4)	1 (5)	1 (3)	
**Treatment outcome**							0.56^a^/0.25^a^
Cured	70 (83)	47 (85)	23 (79)	2 (2)	2 (6)	0	
Death	–	–	–	7 (8)	1 (3)	6 (12)	
LTFU	4 (5)	2 (4)	2 (7)	5 (6)	1 (3)	4 (8)	
Completed	–	–	–	64 (77)	27 (82)	37 (74)	
Not evaluated	9 (11)	6 (11)	3 (10)	5 (6)	2 (2)	3 (6)	
Failure	1 (1)	0	1 (3)	–	–	–	

Values are number (percentage) unless otherwise indicated. Percentages may not total 100% because of rounding or missing data

*ART* antiretroviral therapy, *HIC* high-resource context (Spain), *HIV* human immunodeficiency virus, *LIC* low-resource context (Mozambique), *LTFU* lost to follow-up, *PHC* primary health care, *TB* tuberculosis

^a^Fisher’s exact test

### Estimated organ dose per single chest radiography

In both settings, organ doses from a single pediatric CXR were low and largely confined to thoracic structures.

In the HIC, mean lung doses ranged from 0.002 mGy cm^2^ to 0.225 mGy cm^2^, decreasing with age. Thyroid, esophagus, and spinal-cord doses were modest, while peripheral organs such as the brain and colon received <0.01 mGy cm^2^. In the LIC, single-exposure organ doses were also low, with mean lung doses of 0.019 mGy cm^2^ (females) and 0.010 mGy cm^2^ (males) among infants, declining with age. Thyroid and heart wall doses remained under 0.01 mGy cm^2^ across groups.

**Fig. 2:**
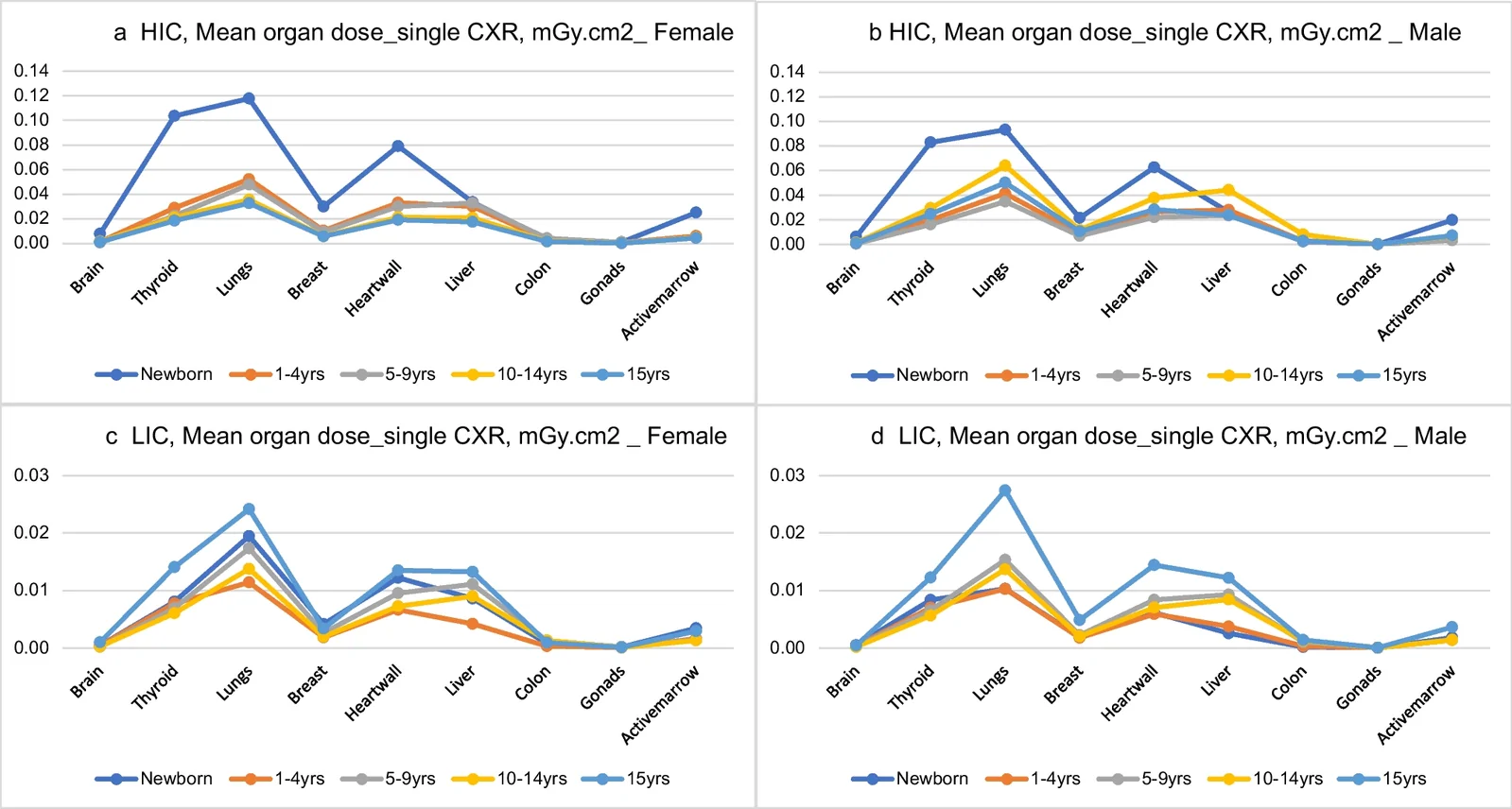
Mean organ dose per single chest radiography (in mGy cm^2^). Mean organ dose per single pediatric chest radiograph, by age group and gender. Each *bar* represents a specific organ (the lungs, thyroid, heart wall, liver, colon, active marrow). Age groups are plotted on the *x*-axis, with organ-specific mean doses (mGy cm^2^) on the *y*-axis. High-income country (**a** and **b**) and low-income country (**c** and **d**) datasets are displayed separately to reflect differences in data acquisition methods

These results confirm that both contexts operate within internationally accepted pediatric exposure ranges, though dose estimation in the LIC relied on standardized assumptions derived from site protocols rather than DICOM data (Fig. 2; Supplementary material 2).

### Cumulative organ doses from routine chest radiography practice

Patterns of imaging use differed markedly between the two contexts.

In HIC, children typically underwent two to six CXRs during TB care, resulting in cumulative thoracic-organ doses that remained below European Diagnostic Reference Levels (EDRLs) [28]. Doses were highest among younger children (mean lung ≈0.8 mGy cm^2^ in newborns) and declined with age. Peripheral-organ exposures were minimal (<0.03 mGy cm^2^).

**Fig. 3:**
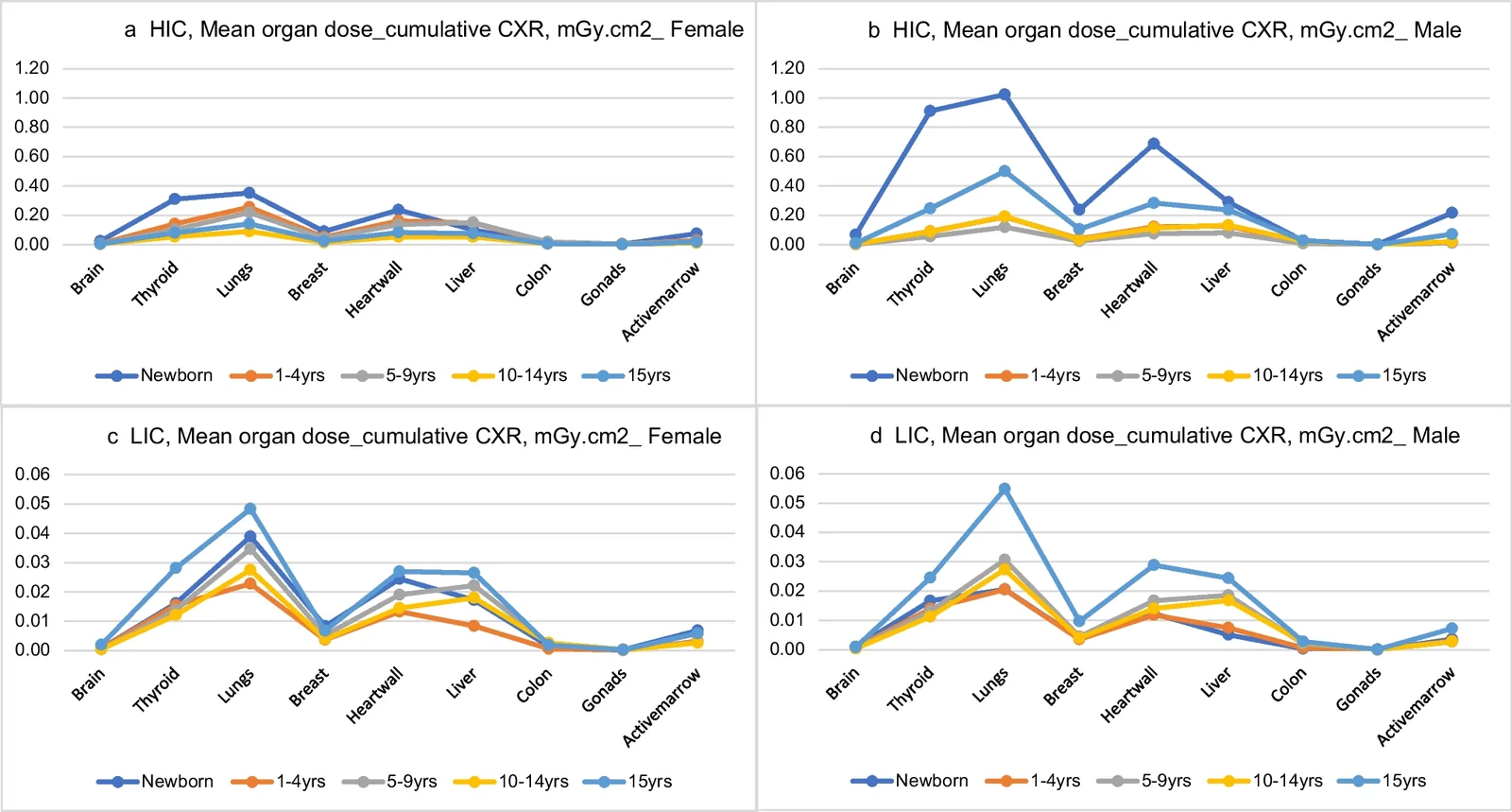
Cumulative organ doses from multiple chest radiographies in routine practice (in mGy cm^2^). Cumulative organ doses from multiple chest radiographies during routine pediatric tuberculosis care, by age group and gender. Each *color-coded series* corresponds to a specific organ (the lungs, thyroid, heart wall, liver, colon, active marrow). Age groups are explicitly labelled on the *x*-axis. Dose values (mGy cm2) represent modelled cumulative exposures based on reported imaging frequency. High-income country (**a** and **b**) and low-income country (**c** and **d**) datasets are displayed separately to reflect differences in data acquisition methods

In the LIC, most children received only one or two CXRs per TB episode, and cumulative thoracic doses stayed below 0.05 mGy cm^2^ across age groups due to limited imaging availability. This very low cumulative exposure coincided with survey-reported constraints in imaging access, including limited on-site X-ray availability, reliance on patient referral for imaging, a predominantly non-radiologist workforce, and the absence of digital archiving systems that would enable longitudinal follow-up or repeat imaging. Infant lung, thyroid, and heart wall doses were generally ≤0.04 mGy cm^2^. The overall magnitude of exposure was extremely low, reflecting limited imaging rather than dose optimization (Fig. 3; Table 3; Supplementary material 2).

### Contribution of computed tomography imaging in the high-resource setting

CT scans were available and routinely used in diagnostically inconclusive cases in the HIC but were not performed in the LIC (neither national nor WHO guidelines recommend the use of CT for TB diagnosis in this context). In the HIC, CT substantially increased total organ doses compared with CXR-only cases. For females aged 10–14 years, lung, heart wall, and breast doses rose from <0.1 mGy cm^2^ (CXR only) to >20 mGy cm^2^ with CT inclusion. In males of similar age, lung doses averaged 11.7 mGy cm^2^ and heart wall 11.8 mGy cm^2^. Across all age groups, CT amplified organ doses by 20–50-fold compared with single-exposure CXR values (Table 2).

**Table 2:** Computed tomography contribution to organ dose (in mGy cm^2^): illustrates the magnitude by which computed tomography increases cumulative organ dose relative to chest radiography alone

Female	Mean dose CXR	Mean dose Total (CXRCT)	Male	Mean dose CXR	Mean dose Total (CXRCT)
**F, newborn**			**M, newborn**		
Brain	0	0	Brain	0.07	0.12
Thyroid	0	0	Thyroid	0.91	2.53
Lungs	0	0	Lungs	1.03	3.05
Breast	0	0	Breast	0.24	1.58
Heart wall	0	0	Heart wall	0.69	2.56
Liver	0	0	Liver	0.29	1.15
Colon	0	0	Colon	0.03	0.08
Active marrow	0	0	Active marrow	0.22	0.73
**F, 1–4 yrs**			**M, 1–4 yrs**		
Brain	0.01	0.06	Brain	0.00	0.05
Thyroid	0.14	1.30	Thyroid	0.09	1.79
Lungs	0.26	3.13	Lungs	0.19	1.70
Breast	0.05	2.57	Breast	0.04	1.28
Heart wall	0.16	3.10	Heart wall	0.12	1.51
Liver	0.15	1.65	Liver	0.13	0.81
Colon	0.01	0.12	Colon	0.01	0.06
Active marrow	0.03	0.62	Active marrow	0.02	0.44
**F, 5–9 yrs**			**M, 5–9 yrs**		
Brain	0.00	0.09	Brain	0.00	0.12
Thyroid	0.10	3.96	Thyroid	0.06	6.69
Lungs	0.22	4.93	Lungs	0.12	5.18
Breast	0.04	3.23	Breast	0.02	2.07
Heart wall	0.14	4.62	Heart wall	0.08	4.28
Liver	0.15	2.26	Liver	0.08	1.62
Colon	0.02	0.13	Colon	0.01	0.11
Active marrow	0.02	1.26	Active marrow	0.01	1.45
**F, 10–14 yrs**			**M, 10–14 yrs**		
Brain	0.00	0.22	Brain	0.00	0.14
Thyroid	0.05	4.85	Thyroid	0.09	6.22
Lungs	0.09	21.14	Lungs	0.19	11.71
Breast	0.02	17.22	Breast	0.04	9.86
Heart wall	0.05	21.23	Heart wall	0.11	11.75
Liver	0.05	20.03	Liver	0.13	8.91
Colon	0.01	1.92	Colon	0.02	0.50
Active marrow	0.01	7.09	Active marrow	0.02	3.45
**F, 15 yrs**			**M, 15 yrs**		
Brain	0.01	0.17	Brain	0.01	0.11
Thyroid	0.08	9.33	Thyroid	0.25	4.26
Lungs	0.14	12.24	Lungs	0.50	10.98
Breast	0.03	9.86	Breast	0.11	9.96
Heart wall	0.08	11.69	Heart wall	0.28	11.16
Liver	0.08	9.73	Liver	0.24	9.65
Colon	0.01	0.63	Colon	0.03	0.71
Active marrow	0.02	3.71	Active marrow	0.07	3.40

Values represent mean absorbed organ dose±standard deviation (minimum–maximum) for patients undergoing chest computed tomography in addition to chest radiography. No computed tomography scans were performed in the low-resource setting. All radiation doses were estimated using National Cancer Institute dosimetry systems for radiography and computed tomography and represent modelled absorbed organ doses rather than directly measured patient exposures

*CT* computed tomography, *CXR* chest radiography, *CXRCT* cumulative chest radiography and computed tomography, *F* female, *M* male

### Cumulative exposure by tuberculosis type and drug resistance category

In HIC, cumulative radiation exposure varied by disease complexity and imaging protocol. Drug-sensitive pulmonary TB showed mean lung doses around 6.6 mGy cm^2^, rising to ≈11 mGy cm^2^ in drug resistant TB and >20 mGy cm^2^ in extrapulmonary TB, reflecting extended scan coverage and repeated CTs.

In the LIC, cumulative doses remained extremely low across all diagnostic categories due to limited imaging availability (<0.03 mGy cm^2^ for pulmonary TB, ≤0.032 mGy cm^2^ for drug resistant or extrapulmonary disease). The low frequency of imaging precluded meaningful differentiation by disease type (Tables 3 and 4).

**Table 3:** Cumulative organ radiation doses from chest radiography among children with tuberculosis, by disease type and drug resistance profile in low-income (Mozambique) and high-income (Spain) settings

Variable	LIC P-sensitive TB	LIC P-resistant TB	LIC EP-sensitive TB	HIC P-sensitive TB	HIC P-resistant/unknown	HIC EP-sensitive TB	HIC EP-resistant/unknown
**Brain**	0.00	0.00	0.00	0.01±0.01 (0.00–0.02)	Res: 0.02±0.04 (0.00–0.09)/Unk: 0.00±0.01 (0–0.04)	0.01 (0.00–0.01)	Res: 0.01 (0.01–0.01)/Unk: 0.02±0.03 (0.00–0.06)
**Thyroid**	0.02±0.01 (0.01–0.03)	0.02±0.02 (0.01–0.02)	0.01±0.01 (0.01–0.03)	0.10±0.09 (0.01–0.4)	Res: 0.28±0.49 (0.04–1.26)/Unk: 0.10±0.10 (0.01–0.56)	0.09 (0.085–0.09)	Res: 0.10 (0.10–0.10)/Unk: 0.34±0.39 (0.10–0.78)
**Lungs**	0.03±0.01 (0.02–0.08)	0.03±0.01 (0.02–0.04)	0.03±0.01 (0.02–0.06)	0.19±0.18 (0.02–0.79)	Res: 0.38±0.52 (0.06–1.41)/Unk: 0.18±0.16 (0.02–0.78)	0.16±0.05 (0.10–0.20)	Res: 0.22 (0.22–0.22)/Unk: 0.47±0.37 (0.22–0.89)
**Breast**	0.01 (0.00–0.02)	0.01 (0.00–0.01)	0.01 (0.00–0.01)	0.04±0.03 (0.00–0.14)	Res: 0.08±0.12 (0.01–0.33)/Unk: 0.04±0.03 (0.00–0.15)	0.03±0.01 (0.03–0.04)	Res: 0.04 (0.04–0.04)/Unk: 0.10±0.10 (0.04–0.21)
**Heart wall**	0.02±0.01 (0.01–0.05)	0.02 (0.01–0.02)	0.02±0.01 (0.01–0.03)	0.12±0.11 (0.01–0.48)	Res: 0.25±0.35 (0.04–0.95)/Unk: 0.12±0.10 (0.01–0.48)	0.10±0.03 (0.07–0.12)	Res: 0.13 (0.13–0.13)/Unk: 0.30±0.30 (0.13–0.59)
**Liver**	0.02±0.01 (0.01–0.04)	0.02±0.01 (0.01–0.02)	0.02±0.01 (0.01–0.02)	0.11±0.11 (0.01–0.51)	Res: 0.16±0.15 (0.04–0.40)/Unk: 0.12±0.10 (0.01–0.51)	0.10±0.06 (0.03–0.14)	Res: 0.16 (0.16–0.16)/Unk: 0.20±0.05 (0.14–0.25)
**Colon**	0.00	0.00	0.00	0.01±0.01 (0.00–0.05)	Res: 0.09±0.01 (0.00–0.04)/Unk: 0.01±0.01 (0.00–0.05)	0.02±0.01 (0.00–0.03)	Res: 0.03 (0.03–0.03)/Unk: 0.02±0.01 (0.01–0.04)
**Active marrow**	0.00 (0.00–0.01)	0.00	0.00 (0.00–0.01)	0.02±0.02 (0.00–0.08)	Res: 0.06±0.12 (0.01–0.30)/Unk: 0.02±0.02 (0.00–0.14)	0.02 (0.02–0.02)	Res: 0.02 (0.02–0.02)/Unk: 0.08±0.09 (0.02–0.19)

Values are expressed as mean±standard deviation (minimum–maximum) absorbed organ dose per tuberculosis category. Low-resource context values are based on standardized site survey parameters, whereas high-resource context values derive from DICOM metadata. Comparisons are descriptive and reflect contextual differences in imaging frequency and modality use rather than clinical outcomes. All radiation doses were estimated using National Cancer Institute dosimetry systems for radiography and represent modelled absorbed organ doses rather than directly measured patient exposures

*EP* extrapulmonary, *HIC* high-resource context (Spain), *LIC* low-resource context (Mozambique), *P* pulmonary, *Res* resistant, *Sens* sensitive, *TB* tuberculosis, *Unk* unknown

**Table 4:** Computed tomography scan cumulative radiation exposure (in mGy cm^2^) by tuberculosis type and resistance profile: presents modelled cumulative organ doses from chest computed tomography examinations in pediatric tuberculosis cases in the high-resource context (Spain)

Variable	HIC P-sensitive	HIC P-resistant/unknown	HIC EP-sens/unknown	HIC+CXR+CT P-sensitive	HIC+CXR+CT P-resistant/unknown	HIC+CXR+CT EP-sens/unknown
**Brain**	0.10±0.07 (0.04–0.20)	Res: 0.18±0.22 (0.02–0.34)/Unk: 0.09±0.11 (0–0.54)	Sens: 0.21 (0.21–0.21)/Unk: 0.22 (0.22–0.22)	0.10±0.07 (0.04–0.20)	Res: 0.23±0.16 (0.12–0.34)/Unk: 0.09±0.11 (0.00–0.54)	Sens: 0.21 (0.21–0.21)/Unk: 0.22 (0.22–0.22)
**Thyroid**	4.07±3.92 (0.34–9.53)	Res: 11.35±14.25 (1.27–21.42)/Unk: 4.07±7.19 (0.09–33.09)	Sens: 4.72 (4.72–4.72)/Unk: 4.85 (4.85–4.85)	4.16±3.95 (0.35–9.87)	Res: 12.00±13.39 (2.53–21.47)/Unk: 4.15±7.17 (0.18–33.10)	Sens: 4.85 (4.85–4.85)/Unk: 4.85 (4.85–4.85)
**Lungs**	6.43±5.84 (1.66–16.59)	Res: 10.44±12.44 (1.64–19.23)/Unk: 4.83±5.34 (0.32–19.95)	Sens: 20.85 (20.85–20.85)/Unk: 21.14 (21.14–21.14)	6.62±5.76 (1.86–16.61)	Res: 11.20±11.53 (3.05–19.36)/Unk: 4.98±5.31 (0.53–20.13)	Sens: 21.14 (21.14–21.14)/Unk: 21.14 (21.14–21.14)
**Breast**	5.33±4.92 (0.10–13.52)	Res: 4.36±4.39 (1.25–7.47)/Unk: 3.42±4.06 (0.09–18.28)	Sens: 17.18 (17.18–17.18)/Unk: 17.22 (17.22–17.22)	5.36±4.90 (0.24–13.53)	Res: 4.53±4.18 (1.58–7.49)/Unk: 3.46±4.05 (0.24–18.31)	Sens: 17.22 (17.22–17.22)/Unk: 17.22 (17.22–17.22)
**Heart wall**	6.37±5.96 (0.68–16.58)	Res: 9.42±11.03 (1.62–17.22)/Unk: 4.53±4.91 (0.33–20.11)	Sens: 21.07 (21.07–21.07)/Unk: 21.23 (21.23–21.23)	6.48±5.90 (1.16–16.59)	Res: 9.93±10.41 (2.56–17.29)/Unk: 4.62±4.89 (0.47–20.22)	Sens: 21.23 (21.23–21.23)/Unk: 21.23 (21.23–21.23)
**Liver**	4.59±4.52 (0.08–12.44)	Res: 2.91±3.04 (0.76–5.06)/Unk: 2.69±3.85 (0.07–16.76)	Sens: 19.83 (19.83–19.83)/Unk: 20.03 (20.03–20.03)	4.71±4.45 (0.59–12.45)	Res: 3.14±2.82 (1.15–5.14)/Unk: 2.78±3.83 (0.14–16.86)	Sens: 20.03 (20.03–20.03)/Unk: 20.03 (20.03–20.03)
**Colon**	0.25±0.25 (0.01–0.71)	Res: 0.19±0.21 (0.04–0.33)/Unk: 0.17±0.24 (0.01–1.13)	Sens: 1.89 (1.89–1.89)/Unk: 1.92 (1.92–1.92)	0.27±0.25 (0.06–0.71)	Res: 0.21±0.19 (0.08–0.34)/Unk: 0.18±0.24 (0.01–1.14)	Sens: 1.92 (1.92–1.92)/Unk: 1.924 (1.92–1.92)
**Active marrow**	1.88±1.76 (0.34–4.89)	Res: 2.55±3.00 (0.43–4.67)/Unk: 1.39±1.73 (0.07–5.74)	Sens: 7.06 (7.06–7.06)/Unk: 7.09 (7.09–7.09)	1.90±1.75 (0.36–4.89)	Res: 2.70±2.79 (0.73–4.68)/Unk: 1.40±1.73 (0.09–5.74)	Sens: 7.09 (7.09–7.09)/Unk: 7.09 (7.09–7.09)

Values are expressed as mean±standard deviation (minimum–maximum) estimated absorbed organ dose. No computed tomography data were available for the low-resource context (Mozambique) cohort. All radiation doses were estimated using National Cancer Institute dosimetry systems for computed tomography and represent modelled absorbed organ doses rather than directly measured patient exposures

*CT* computed tomography, *CXR* chest radiography, *EP* extrapulmonary, *HIC* high-resource context (Spain), *LIC* low-resource context (Mozambique), *P* pulmonary, *Res* resistant, *Sens* sensitive, *Unk* unknown

## Discussion

This study provides one of the first descriptive assessments of pediatric radiation exposure and imaging practices for TB across contrasting healthcare contexts. By documenting both estimated organ doses and system-level practices in Spain and Mozambique, the findings illustrate how health-system capacity, infrastructure, and human resources influence imaging use patterns and the resulting cumulative radiation doses in children being evaluated for TB (Table 5).

**Table 5:** Health-system contexts shaping pediatric tuberculosis imaging practices and radiation exposure

High-resource context (Spain)	Low-resource context (Mozambique)
**Service level:** tertiary hospitals with integrated radiology departments	**Service level:** primary health centers with limited imaging infrastructure
**Imaging availability:** CXR and CT onsite; digital PACS archiving	**Imaging availability:** CXR available at selected sites; no CT scanners; paper or manual logs
**Human resources:** radiologists and trained radiographers; pediatric-specific protocols	**Human resources:** non-physician clinicians interpret CXRs; limited radiology training
**Dose monitoring:** DICOM-based dose recording; national DRLs and QA systems	**Dose monitoring:** no routine dose tracking; no local DRLs
**Typical practice:** multiple CXRs and CTs primarily for diagnostically inconclusive or complex cases	**Typical practice:** one or two CXRs per episode; minimal follow-up imaging
**Cumulative radiation:** slightly higher cumulative dose (due to CT use), but optimized	**Cumulative radiation:** extremely low doses, reflecting restricted imaging access
**Diagnostic adequacy:** high, supported by imaging quality and radiologist interpretation	**Diagnostic adequacy:** variable, limited by equipment, training, and image quality
**Equity implication:** continuous dose optimization	**Equity implication:** need to strengthen infrastructure, QA, and pediatric DRLs

Schematic summary of contextual differences influencing pediatric tuberculosis imaging practices and radiation exposure in Spain (high-resource context) and Mozambique (low-resource context)

*CT* computed tomography, *CXR* chest radiography, *DICOM* digital imaging and communications in medicine, *DRL* diagnostic reference level, *PACS* picture archiving and communication system, *QA* quality assurance

In both settings, radiation doses from single and cumulative CXRs were low and within internationally accepted limits. In the HIC, mean DAP values (1.6–6.9.6.9 mGy cm^2^) were well below the 2018 European Diagnostic Reference Levels (EDRLs) (22–87 mGy cm^2^) and national DRLs (40–100 mGy cm^2^) [28]. This reflects established pediatric protocols, radiographer training, and quality assurance mechanisms designed to maintain diagnostic adequacy while minimizing exposure. In the LIC, DAP values (0.5–6.2.5.2 mGy cm^2^) were also low but derived from standardized assumptions rather than direct DICOM data, underscoring the importance of strengthening dose documentation systems in low-resource environments.

CT contributed most to cumulative dose in the high-resource setting. Although its use markedly increased organ doses, particularly to the lungs, thyroid, and heart wall, absolute exposures remained well below thresholds associated with deterministic effects. In this study, CT was primarily performed in diagnostically inconclusive pediatric TB cases where conventional radiography and clinical evaluation were insufficient to confirm or exclude disease. These findings are consistent with international evidence that CT, when clinically justified, plays a critical role in evaluating complex or uncertain pediatric TB presentations [5, 29–31].

This aligns with recent pediatric TB imaging recommendations, which outline clear decision-making algorithms and emphasize limiting CT use to cases where it meaningfully enhances diagnostic confidence. Adherence to justification principles and the ALARA framework remains essential, given the higher relative doses involved [6]. In some high-resource settings, chest MRI has been explored as a radiation-free alternative to CT for evaluating mediastinal lymphadenopathy in children, although its limited availability, longer acquisition times, and lack of integration into current pediatric TB imaging guidelines restrict its routine use [9, 18, 32, 33].

Importantly, the quantitative dose patterns observed in both settings closely aligned with the contextual constraints identified in the surveys. In the LIC, the extremely low cumulative radiation doses observed in the LIC cohort should not be interpreted as evidence of dose optimization. Instead, these findings directly reflect structural constraints identified in the accompanying surveys (limited imaging availability, workforce shortages, and lack of archiving) which collectively restrict imaging frequency and continuity of care. Survey findings showed that 87% of CXRs were interpreted by non-radiologist clinicians and that 37% of providers had never received formal CXR training, reducing confidence in interpretation and limiting justification for repeat imaging. As a consequence, clinicians tended to request only one or two CXRs per TB episode, even when follow-up imaging might have improved diagnostic certainty. This directly shaped the extremely low cumulative doses detected in the quantitative analysis.

In contrast in the HIC, the use of multiple CXRs and CT scans reflects the availability of imaging infrastructure and trained radiologists who can justify advanced imaging. Survey findings show routine dose monitoring, pediatric protocols, and radiologist oversight, enabling both higher imaging frequency and safer, optimized exposure. Therefore, higher CT-related doses occur within a system that systematically justifies and monitors imaging. These results highlight that radiation exposure patterns are closely tied to workforce capacity, infrastructure, and quality assurance systems. In contrast, limited radiology training and equipment in the LIC restrict imaging access and shape diagnostic pathways and cumulative dose.

In low-resource settings, the pattern of minimal cumulative radiation exposure must be interpreted within a broader context of diagnostic system performance. Limited image archiving and weak quality assurance mechanisms restrict longitudinal assessment and reduce opportunities for structured follow-up imaging, while variable adherence to imaging guidelines and dependence on manual exposure settings potentially introduce heterogeneity in image quality. Previous assessments have reported similar challenges, including lack of national DRLs and minimal quality assurance structures [34–36]. In such environments, strengthening training, standardizing protocols, and introducing dose monitoring tools are key priorities for improving pediatric imaging practice.

While the risk of radiation-induced harm was minimal in both settings, the broader concern is diagnostic equity. Children in high-resource environments benefit from structured imaging pathways, radiologist oversight, and access to advanced modalities, ensuring timely and accurate diagnosis. In low-resource primary care systems with limited radiological capacity, previous studies have reported that under-imaging may contribute to delayed recognition of subtle pediatric TB findings; although our study did not assess diagnostic delay directly, this remains an important contextual consideration in interpreting imaging access in Mozambique [37–40]. Aligning with the WHO and the International Commission on Radiological Protection (ICRP) recommendations, dose optimization should therefore aim not only to reduce exposure but also to ensure adequate image quality for confident diagnosis [15, 16, 28, 29].

This study also highlights the value of implementing pediatric-specific imaging protocols and establishing locally relevant DRLs. Even in settings with minimal equipment, developing standard exposure charts, documenting DAP values, and training radiographers in pediatric dose adjustment can improve both safety and diagnostic reliability. Digital image storage systems, even at basic regional hospitals, could facilitate longitudinal assessment and quality control. In high-resource environments, the focus should remain on reducing CT utilization where possible, using lower-dose protocols or alternative modalities such as ultrasound for extrapulmonary disease and treatment follow-up [39, 41, 42].

The site selection in both countries reflects how pediatric TB care is routinely organized and is therefore unlikely to introduce selection bias. In Spain, children with suspected TB are typically managed in tertiary hospitals with pediatric infectious diseases and pediatric radiology services, making the participating centers representative of routine care. In Mozambique, pediatric TB diagnosis occurs mainly in primary care facilities, and the selected health centers therefore reflect standard service delivery in this context. As such, the observed differences reflect true contextual contrasts rather than artifacts of sampling.

This study has several limitations. Radiation dose data differed in precision between settings: DICOM-based, patient-specific estimates were possible in the HIC, whereas LIC estimates relied on standardized protocols and survey inputs, introducing uncertainty but not altering the finding of very low cumulative doses due to infrequent imaging. As a retrospective study, selection bias is possible, and we could not adjust fully for disease severity, which may have contributed to higher CT use in the HIC. Generalizability is limited, as imaging availability, protocols, and workforce vary across countries. Important CXR technique parameters could not be assessed, especially in the LIC, and may account for unmeasured dose variation. CT use in the HIC, although consistent with guideline-based justification for complex or inconclusive cases, remains the main contributor to cumulative dose, but we did not systematically document clinical indications or evaluate patient-level outcomes. We were also unable to explore associations between cumulative dose and morbidity, mortality, or long-term follow-up due to inconsistent outcome documentation across settings. Future prospective, harmonized studies are needed to address these gaps. Despite these limitations, the convergence of dose estimates with survey findings supports the overall interpretation.

## Conclusion

This study shows that differences in pediatric TB imaging practices reflect disparities in resources, infrastructure, and workforce capacity rather than clinical approach. Because dose alone does not indicate diagnostic adequacy, access to high-quality and appropriately justified imaging depends heavily on the health-system context. In low-resource settings, very low cumulative doses stem from limited imaging availability, highlighting the need to strengthen infrastructure, implement pediatric exposure charts and local DRLs, expand radiology training for non-physician clinicians, and introduce basic dose monitoring and archiving systems. In high-resource settings, where CT drives higher cumulative doses, reinforcing dose monitoring, reviewing justification criteria, and adopting lower-dose or radiation-free alternatives such as ultrasound can further protect children. Overall, context-specific strategies that improve radiation protection, workforce capacity, and quality assurance are essential to advance safe, equitable, and reliable pediatric TB imaging across diverse settings.

## Supplementary Information

Below is the link to the electronic supplementary material.

Supplementary file1 (DOCX 25.5 KB)

Supplementary file2 (DOCX 45.1 KB)

## Data Availability

No datasets were generated or analysed during the current study.
