# Supplementary material for: Contextual dimensions of pediatric tuberculosis imaging: radiation exposure, access, and system capacity in high- and low-resource settings
Source: Pediatr Radiol. 2026 Feb 18;56(4):936–50. doi: 10.1007/s00247-026-06535-z (PMC13035884; doi:10.1007/s00247-026-06535-z)
Supplement: Supplementary file 1 — Supplementary file1 (DOCX 25.5 KB) [file 247_2026_6535_MOESM1_ESM.docx]

**Supplementary material 1: comparative survey on imaging use for pediatric tuberculosis in LIC setting (Mozambique) and HIC setting (Spain)**

**Supplemental table 1. Demographic characteristics of survey respondents from low-income (LIC, Mozambique) and high-income (HIC, Spain) settings.**

| **Variable** | **LIC**  **n=68 (%)** | **HIC**  **n=36 (%)** |
| --- | --- | --- |
| **Health facility type** | 61 primary health centres (90%)  4 hospitals (6%)  3 others (4%) | 36 hospitals (100%) |
| **Age** | 20 - 34: 39 (57%)  35 - 49: 26 (38%)  50 - 64: 3 (4%)  65+: 0 (0%) | 20 - 34: 2 (6%)  35 - 49: 22 (61%)  50 - 64: 10 (28%)  65+: 2 (6%) |
| **Sex** | Female: 44 (65%)  Male: 24 (35%) | Female: 23 (64%)  Male: 13 (36%) |
| **Staff category** | Non-doctor clinicians: 60 (88%)  Doctors: 8 (12%) | Doctors: 36 (100%) |
| **Years at facility** | <15 years: 64 (94%)  15 - 30 years: 4 (6%)  >30 years: 0 (0%) | <15 years: 16 (44%)  15 - 30 years: 17 (47%)  >30 years: 3 (8%) |
| **Years in current role** | <15 years: 61 (90%)  15 - 30 years: 7 (10%)  >30 years: 0 (0%) | <15 years: 12 (33%)  15 - 30 years: 21 (58%)  >30 years: 3 (8%) |

**Supplemental table 2. Comparison of imaging access and turnaround times for chest x-ray (CXR) and computed tomography (CT) between LIC and HIC facilities.**

| **Item** | **LIC**  **n=68 unless noted (%)** | **HIC**  **n=36 (%)** |
| --- | --- | --- |
| **CXR onsite access** | 23 (34%) | 36 (100%) |
| **Transferred for CXR** | 36 (53%) | 0 (0%) |
| **No access to CXR** | 9 (13%) | 0 (0%) |
| **CT scan onsite access** |  | 34 (94%) |
| **Transferred for CT scan** |  | 2 (6%) |
| **No access to CT scan** |  | 0 (0%) |
| **Time to obtain CXR** | Same day: 50 (74%)  <1 week: 15 (22%)  >1 week: 3 (4%) | Same day: 35 (97%)  <1 week: 1 (3%)  >1 week: 0 (0%) |
| **Time to obtain CT scan** |  | Same day: 9 (25%)  <1 week: 23 (64%)  >1 week: 9 (25%) |

**Supplemental table 3. Availability of local imaging guidelines and proportion of presumptive tuberculosis (TB) patients undergoing pre-consultation imaging in LIC and HIC settings.**

| **Item** | **LIC**  **n=68 (%)** | **HIC**  **n=36 (%)** |
| --- | --- | --- |
| **Local CXR guideline available** | 55 (81%) | 36 (100%) |
| **Presumptive TB patients with prior CXR** | <30%: 49 (72%)  30 - 60%: 12 (18%)  >60%: 7 (10%) | <30%: 17 (47%)  30 - 60%: 5 (14%)  >60%: 14 (39%) |
| **Presumptive TB patients with prior CT scan** |  | <30%: 35 (97%)  30 - 60%: 0 (0%)  >60%: 1 (3%) |

**Supplemental table 4. Roles of different healthcare cadres in ordering and interpreting chest x-ray (CXR) and CT scans across LIC and HIC facilities.**

| **Role** | **LIC**  **n=68 (%)** | **HIC**  **n=36 (%)** |
| --- | --- | --- |
| CXR ordered by medical doctors | 37 (54%) | 36 (100%) |
| CXR ordered by non-doctor clinicians | 52 (76%) | 3 (8%) |
| CXR ordered by radiology staff | 1 (1%) | 2 (6%) |
| CXR requested by patient/guardian | 24 (35%) | 0 (0%) |
| CT scan ordered by medical doctors |  | 36 (100%) |
| CT scan ordered by radiology staff |  | 16 (44%) |
| CXR interpreted by medical doctors | 54 (79%) | 22 (61%) |
| CXR interpreted by non-doctor clinicians | 59 (87%) | 0 (0%) |
| CXR interpreted by radiology staff | 5 (7%) | 35 (97%) |
| CXR interpreted remotely | 0 (0%) | 1 (3%) |
| CT interpreted by medical doctors |  | 6 (17%) |
| CT interpreted by radiology staff |  | 36 (100%) |
| CT interpreted remotely |  | 0 (0%) |

**Supplemental table 5. Levels of confidence and training among respondents in interpreting chest x-rays and CT scans in LIC and HIC settings.**

| **Item** | **LIC**  **n=68 (%)** | **HIC**  **n=36 (%)** |
| --- | --- | --- |
| Confident reading CXR | 30 (44%) | 10 (28%) |
| Moderately confident reading CXR | 36 (53%) | 25 (69%) |
| Not confident reading CXR | 2 (3%) | 1 (3%) |
| Formal CXR course | 14 (21%) | 5 (14%) |
| CXR training at medical school | 16 (24%) | 17 (47%) |
| CXR clinical rounds | 18 (26%) | 26 (72%) |
| No CXR training | 25 (37%) | 0 (0%) |
| Formal CT course |  | 1 (3%) |
| CT training at medical school |  | 14 (39%) |
| CT clinical rounds |  | 18 (50%) |
| No CT training |  | 7 (19%) |

**Supplemental table 6. Perceptions of radiation risk associated with chest x-ray, CT, and ultrasound among respondents in LIC and HIC settings.**

| **Factor** | **LIC**  **n=68 (%)** | **HIC**  **n=36 (%)** |
| --- | --- | --- |
| Perceivex-ray as risky | 54 (79%) | 26 (72%) |
| Perceive CT as risky |  | 34 (94%) |
| Perceive ultrasound as risky | 37 (54%) | 0 (0%) |
| Consider radiation exposure when ordering CXR | 34 (50%) | 27 (75%) |
| Consider radiation exposure when ordering CT |  | 33 (92%) |
| Consider radiation exposure when ordering ultrasound | 20 (29%) | 9 (25%) |

**Supplemental table 7. Radiation knowledge (C = correct answer)**

**Supplemental table 7A. Estimated average radiation dose (mSv) of a standard pediatric chest x-ray among respondents from LIC and HIC settings.**

(Q: What is your estimate of the average radiation dose of a standard chest radiograph applied to a child?)

| **Dose estimate** | **LIC**  **n=68 (%)** | **HIC**  **n=36 (%)** |
| --- | --- | --- |
| <0.01 mSv | 2 (3%) | 2 (6%) |
| **0.01–<0.1 mSv (C)** | 2 (3%) | 6 (17%) |
| 0.1–<1 mSv | 3 (4%) | 7 (19%) |
| 1–<10 mSv | 5 (7%) | 2 (6%) |
| 10–<100 mSv | 0 (0%) | 1 (3%) |
| >100 mSv | 0 (0%) | 0 (0%) |
| Don’t know | 56 (82%) | 18 (50%) |

**Supplemental table 7B. Estimated radiation dose of a pediatric chest CT scan relative to a chest x-ray (CXR = 1 unit) among respondents from LIC and HIC settings. (Relative Dose Ratio)**

(Q: If you consider the effective dose of an X-ray chest examination in a child to be one unit - how many equivalent units do you estimate the chest CT in a child without dose adjustment?)

| **Ratio estimate** | **LIC**  **n=68 (%)** | **HIC**  **n=36 (%)** |
| --- | --- | --- |
| <1x (lower) | 2 (3%) | 0 (0%) |
| 1x (equal) | 3 (4%) | 0 (0%) |
| 2–10x higher | 7 (10%) | 1 (3%) |
| 11–100x higher | 2 (3%) | 16 (44%) |
| **101–1000x higher (C)** | 0 (0%) | 12 (33%) |
| >1000x higher | 1 (1%) | 0 (0%) |
| Don’t know | 53 (78%) | 7 (19%) |

**Supplemental table 7C. Estimated excess lifetime cancer risk from a single CT scan in children under 10 years old, as reported by respondents from LIC and HIC settings.**

(Q: The current estimate of the excess lifetime cancer risk of a child below 10 undergoing any CT scan is approximately:)

| **Risk estimate** | **LIC**  **n=68 (%)** | **HIC**  **n=36 (%)** |
| --- | --- | --- |
| No excess risk | 3 (4%) | 1 (3%) |
| 1 case in 100,000 | 4 (6%) | 6 (17%) |
| 1 case in 10,000 | 4 (6%) | 6 (17%) |
| **1 case in 5,000 (C)** | 1 (1%) | 0 (0%) |
| **1 case in 1,000 (C)** | 5 (7%) | 2 (6%) |
| Don’t know | 51 (75%) | 21 (58%) |

**Supplemental table 7D. Perceived radiation sensitivity of selected organs and systems among respondents from LIC and HIC settings.**

| **Organ/system** | **Sensitivity level** | **LIC**  **n=68 (%)** | **HIC**  **n=36 (%)** |
| --- | --- | --- | --- |
| **Bone Marrow** | Low | 9 (13%) | 2 (6%) |
|  | Medium | 22 (32%) | 9 (25%) |
|  | **High (C)** | 13 (19%) | 20 (56%) |
|  | Don’t know | 24 (35%) | 5 (14%) |
| **Gonads** | Low | 13 (19%) | 0 (0%) |
|  | Medium | 9 (13%) | 3 (8%) |
|  | **High (C)** | 20 (29%) | 31 (86%) |
|  | Don’t know | 26 (38%) | 2 (6%) |
| **Endocrine** | Low | 10 (15%) | 2 (6%) |
|  | **Medium (C)** | 22 (32%) | 12 (33%) |
|  | High | 9 (13%) | 20 (56%) |
|  | Don’t know | 27 (40%) | 2 (6%) |
| **Brain/CNS** | **Low (C)** | 7 (10%) | 5 (14%) |
|  | Medium | 21 (31%) | 20 (56%) |
|  | High | 14 (21%) | 7 (19%) |
|  | Don’t know | 26 (38%) | 4 (11%) |
