# Supplementary material for: Contextual dimensions of pediatric tuberculosis imaging: radiation exposure, access, and system capacity in high- and low-resource settings
Source: Pediatr Radiol. 2026 Feb 18;56(4):936–50. doi: 10.1007/s00247-026-06535-z (PMC13035884; doi:10.1007/s00247-026-06535-z)
Supplement: Supplementary file 2 — Supplementary file2 (DOCX 45.1 KB) [file 247_2026_6535_MOESM2_ESM.docx]

**Supplementary material 2:**

**Supplemental table 8a: Measured dose–area product (DAP) values (Gy·cm²) obtained from image files in the high-income-country (HIC) cohort, disaggregated by gender and age group.**

| **HIC setting DAP values (from image files) per gender and age group** | | | | |
| --- | --- | --- | --- | --- |
| **DAP_female** | **Mean** | **Std. dev** | **Min** | **Max** |
| **Newborn** | 0.02 | 0.02 | 0.01 | 0.09 |
| **1-4yrs** | 0.04 | 0.03 | 0.02 | 0.17 |
| **5-9yrs** | 0.04 | 0.03 | 0.01 | 0.11 |
| **10-14yrs** | 0.03 | 0.02 | 0.01 | 0.06 |
| **15+yrs** | 0.04 | 0.02 | 0.01 | 0.08 |
| **DAP_male** | **Mean** | **Std. dev** | **Min** | **Max** |
| **Newborn** | 0.02 | 0.01 | 0.01 | 0.02 |
| **1-4yrs** | 0.05 | 0.04 | 0.01 | 0.23 |
| **5-9yrs** | 0.04 | 0.03 | 0.01 | 0.18 |
| **10-14yrs** | 0.07 | 0.06 | 0.01 | 0.32 |
| **15+yrs** | 0.07 | 0.07 | 0.02 | 0.19 |

**Supplemental table 8b: Estimated dose–area product (DAP) values (Gy·cm²) for the low-income-country (LIC) cohort, modeled per gender and age group.**

| **Estimated LIC setting DAP values per gender and age group** | | | | |
| --- | --- | --- | --- | --- |
| **DAP_female** | **Mean** | **Std. dev** | **Min** | **Max** |
| **Newborn** | 0.01 | 0.00 | 0.01 | 0.01 |
| **1-4yrs** | 0.01 | 0.00 | 0.01 | 0.01 |
| **5-9yrs** | 0.02 | 0.00 | 0.01 | 0.02 |
| **10-14yrs** | 0.02 | 0.00 | 0.02 | 0.02 |
| **15+yrs** | 0.06 | 0.00 | 0.06 | 0.06 |
| **DAP_male** | **Mean** | **Std. dev** | **Min** | **Max** |
| **Newborn** | 0.01 | 0.00 | 0.01 | 0.01 |
| **1-4yrs** | 0.01 | 0.00 | 0.01 | 0.02 |
| **5-9yrs** | 0.02 | 0.01 | 0.01 | 0.02 |
| **10-14yrs** | 0.02 | 0.00 | 0.02 | 0.02 |
| **15+yrs** | 0.06 | 0.00 | 0.06 | 0.06 |

**Supplemental table 8c: Estimated *HVL* values by gender, age group and context**

|  | **High-income context** | | | | **Low-income context** | | | |
| --- | --- | --- | --- | --- | --- | --- | --- | --- |
| **HVL_Female_Agegroup** | **Mean** | **Std. dev** | **Min** | **Max** | **Mean** | **Std. dev** | **Min** | **Max** |
| **Newborn** | 4.08 | 0.45 | 3.01 | 4.53 | 2.25 | 0.00 | 2.25 | 2.25 |
| **1-4yrs** | 4.10 | 0.46 | 3.01 | 4.53 | 2.30 | 0.14 | 2.25 | 2.61 |
| **5-9yrs** | 4.12 | 0.59 | 1.89 | 4.53 | 2.61 | 0.00 | 2.61 | 2.61 |
| **10-14yrs** | 4.16 | 0.38 | 3.75 | 4.53 | 2.61 | 0.00 | 2.61 | 2.61 |
| **15+yrs** | 4.42 | 0.28 | 3.75 | 4.53 | 3.38 | 0.00 | 3.38 | 3.38 |
| **HVL_Male_Agegroup** |  |  |  |  |  |  |  |  |
| **Newborn** | 4.13 | 0.39 | 3.75 | 4.53 | 2.25 | 0.00 | 2.25 | 2.25 |
| **1-4yrs** | 4.08 | 0.58 | 1.89 | 4.53 | 2.27 | 0.09 | 2.25 | 2.61 |
| **5-9yrs** | 4.07 | 0.51 | 2.61 | 4.53 | 2.55 | 0.14 | 2.25 | 2.61 |
| **10-14yrs** | 3.86 | 0.40 | 3.01 | 4.53 | 2.61 | 0.00 | 2.61 | 2.61 |
| **15+yrs** | 4.14 | 0.39 | 3.75 | 4.53 | 3.38 | 0.00 | 3.38 | 3.38 |

**Supplemental table 8d: Estimated chest radiography parameters in low-income context by age group**

| **Age Group** | **Phantom age year** | **Average Field Width (cm)** | **Average Field Height (cm)** | **ISOX** | **ISOY** | **ISOZ** |
| --- | --- | --- | --- | --- | --- | --- |
| **Newborn** | **1** | 10 | 10 | 12.3 | 8.7 | 30.5 |
| **1–4 years** | **2** | 18 | 18 | 19.3 | 9.3 | 56.6 |
| **5–9 years** | **3** | 22 | 22 | 26.9 | 13.8 | 80.2 |
| **10–14 years** | **4** | 26 | 32 | 35.1 | 14.2 | 105.7 |
| **15 years** | **5** | 30 | 36 | 44.8 | 23 | 127.9 |

**Supplemental table 8e: Extracted chest radiography parameters in high-income context by age group**

| **Age Group** | **Phantom age year** | **Average Field Width (cm)** | **Average Field Height (cm)** | **ISOX** | **ISOY** | **ISOZ** |
| --- | --- | --- | --- | --- | --- | --- |
| **Newborn** | **1** |  |  |  |  |  |
| **1–4 years** | **2** | 24 | 30 | 23.25 | 10.9 | 62.35 |
| **5–9 years** | **3** | 30 | 30 | 35.1 | 13.9 | 104 |
| **10–14 years** | **4** | 35 | 40 | 44.4 | 16.1 | 129.8 |
| **15 years** | **5** | 35 | 43 | 44.4 | 16.1 | 129.8 |

**Supplemental table 8f: Computed tomography parameters in high-income context (kVp, mA, CTDIvol) by age group**

| **Age group** | **kVp** | **Exposure (mA)** | **CTDIvol(mGy)** |
| --- | --- | --- | --- |
| **newborn** | 80 | 63 - 100 | 0.9 - 1.9 |
| **1-4yrs** | 80 - 120 | 40 - 150 | 0.015 - 9.8 |
| **5-9yrs** | 81 - 120 | 19.1 - 150 | 0.017 - 9.8 |
| **10-14yrs** | 80 - 120 | 60 - 224 | 0.015 - 17.5 |

**Supplemental table 9: Mean organ dose (mGy·cm²) per single pediatric chest X-ray (CXR) in HIC and LIC cohorts, stratified by gender and age group.**

| **Mean_HIC cohort, female** | **Newborn** | **1-4yrs** | **5-9yrs** | **10-14yrs** | **15yrs** |  | **Mean_HIC cohort, male** | **Newborn** | **1-4yrs** | **5-9yrs** | **10-14yrs** | **15yrs** |
| --- | --- | --- | --- | --- | --- | --- | --- | --- | --- | --- | --- | --- |
| Brain | 0.01 | 0.00 | 0.00 | 0.00 | 0.00 |  | Brain | 0.01 | 0.00 | 0.00 | 0.00 | 0.00 |
| Thyroid | 0.10 | 0.03 | 0.02 | 0.02 | 0.02 |  | Thyroid | 0.08 | 0.02 | 0.02 | 0.03 | 0.03 |
| Lungs | 0.12 | 0.05 | 0.05 | 0.04 | 0.03 |  | Lungs | 0.09 | 0.04 | 0.04 | 0.06 | 0.05 |
| Breast | 0.03 | 0.01 | 0.01 | 0.01 | 0.01 |  | Breast | 0.02 | 0.01 | 0.01 | 0.01 | 0.01 |
| Heartwall | 0.08 | 0.03 | 0.03 | 0.02 | 0.02 |  | Heartwall | 0.06 | 0.03 | 0.02 | 0.04 | 0.03 |
| Liver | 0.03 | 0.03 | 0.03 | 0.02 | 0.02 |  | Liver | 0.03 | 0.03 | 0.02 | 0.04 | 0.02 |
| Colon | 0.00 | 0.00 | 0.00 | 0.00 | 0.00 |  | Colon | 0.00 | 0.00 | 0.00 | 0.01 | 0.00 |
| Gonads | 0.00 | 0.00 | 0.00 | 0.00 | 0.00 |  | Gonads | 0.00 | 0.00 | 0.00 | 0.00 | 0.00 |
| Activemarrow | 0.03 | 0.01 | 0.00 | 0.01 | 0.00 |  | Activemarrow | 0.02 | 0.00 | 0.00 | 0.01 | 0.01 |
|  |  |  |  |  |  |  |  |  |  |  |  |  |
| **Mean_LIC cohort, female** | **Newborn** | **1-4yrs** | **5-9yrs** | **10-14yrs** | **15yrs** |  | **Mean_LIC cohort, male** | **Newborn** | **1-4yrs** | **5-9yrs** | **10-14yrs** | **15yrs** |
| Brain | 0.00 | 0.00 | 0.00 | 0.00 | 0.00 |  | Brain | 0.00 | 0.00 | 0.00 | 0.00 | 0.00 |
| Thyroid | 0.01 | 0.01 | 0.01 | 0.01 | 0.01 |  | Thyroid | 0.01 | 0.01 | 0.01 | 0.01 | 0.01 |
| Lungs | 0.02 | 0.01 | 0.02 | 0.01 | 0.02 |  | Lungs | 0.01 | 0.01 | 0.02 | 0.01 | 0.03 |
| Breast | 0.00 | 0.00 | 0.00 | 0.00 | 0.00 |  | Breast | 0.00 | 0.00 | 0.00 | 0.00 | 0.01 |
| Heartwall | 0.01 | 0.01 | 0.01 | 0.01 | 0.01 |  | Heartwall | 0.01 | 0.01 | 0.01 | 0.01 | 0.01 |
| Liver | 0.01 | 0.00 | 0.01 | 0.01 | 0.01 |  | Liver | 0.00 | 0.00 | 0.01 | 0.01 | 0.01 |
| Colon | 0.00 | 0.00 | 0.00 | 0.00 | 0.00 |  | Colon | 0.00 | 0.00 | 0.00 | 0.00 | 0.00 |
| Gonads | 0.00 | 0.00 | 0.00 | 0.00 | 0.00 |  | Gonads | 0.00 | 0.00 | 0.00 | 0.00 | 0.00 |
| Activemarrow | 0.00 | 0.00 | 0.00 | 0.00 | 0.00 |  | Activemarrow | 0.00 | 0.00 | 0.00 | 0.00 | 0.00 |

**Supplemental table 10: Cumulative organ doses (mGy·cm²) from multiple chest X-rays performed during routine tuberculosis management in HIC and LIC cohorts, stratified by gender and age group.**

| **Mean_HIC cohort, female** | **Newborn** | **1-4yrs** | **5-9yrs** | **10-14yrs** | **15yrs** |  | **Mean_HIC cohort, male** | **Newborn** | **1-4yrs** | **5-9yrs** | **10-14yrs** | **15yrs** |
| --- | --- | --- | --- | --- | --- | --- | --- | --- | --- | --- | --- | --- |
| Brain | 0.02 | 0.01 | 0.00 | 0.00 | 0.01 |  | Brain | 0.07 | 0.00 | 0.00 | 0.00 | 0.01 |
| Thyroid | 0.31 | 0.14 | 0.10 | 0.05 | 0.08 |  | Thyroid | 0.91 | 0.09 | 0.06 | 0.09 | 0.25 |
| Lungs | 0.35 | 0.27 | 0.22 | 0.09 | 0.14 |  | Lungs | 1.03 | 0.19 | 0.12 | 0.19 | 0.50 |
| Breast | 0.09 | 0.05 | 0.04 | 0.02 | 0.03 |  | Breast | 0.24 | 0.04 | 0.02 | 0.04 | 0.11 |
| Heartwall | 0.24 | 0.16 | 0.14 | 0.05 | 0.08 |  | Heartwall | 0.69 | 0.12 | 0.08 | 0.11 | 0.28 |
| Liver | 0.10 | 0.15 | 0.15 | 0.05 | 0.08 |  | Liver | 0.29 | 0.13 | 0.08 | 0.13 | 0.24 |
| Colon | 0.01 | 0.01 | 0.02 | 0.01 | 0.01 |  | Colon | 0.03 | 0.01 | 0.01 | 0.02 | 0.03 |
| Gonads | 0.00 | 0.00 | 0.00 | 0.00 | 0.00 |  | Gonads | 0.00 | 0.00 | 0.00 | 0.00 | 0.00 |
| Activemarrow | 0.08 | 0.03 | 0.02 | 0.01 | 0.02 |  | Activemarrow | 0.22 | 0.02 | 0.01 | 0.02 | 0.07 |
|  |  |  |  |  |  |  |  |  |  |  |  |  |
| **Mean_LIC cohort, female** | **Newborn** | **1-4yrs** | **5-9yrs** | **10-14yrs** | **15yrs** |  | **Mean_LIC cohort, male** | **Newborn** | **1-4yrs** | **5-9yrs** | **10-14yrs** | **15yrs** |
| Brain | 0.00 | 0.00 | 0.00 | 0.00 | 0.00 |  | Brain | 0.00 | 0.00 | 0.00 | 0.00 | 0.00 |
| Thyroid | 0.02 | 0.02 | 0.01 | 0.01 | 0.03 |  | Thyroid | 0.02 | 0.01 | 0.01 | 0.01 | 0.03 |
| Lungs | 0.04 | 0.02 | 0.04 | 0.03 | 0.05 |  | Lungs | 0.02 | 0.02 | 0.03 | 0.03 | 0.06 |
| Breast | 0.01 | 0.00 | 0.01 | 0.00 | 0.01 |  | Breast | 0.00 | 0.00 | 0.00 | 0.00 | 0.01 |
| Heartwall | 0.02 | 0.01 | 0.02 | 0.02 | 0.03 |  | Heartwall | 0.01 | 0.01 | 0.02 | 0.01 | 0.03 |
| Liver | 0.02 | 0.01 | 0.02 | 0.02 | 0.03 |  | Liver | 0.01 | 0.01 | 0.02 | 0.02 | 0.02 |
| Colon | 0.00 | 0.00 | 0.00 | 0.00 | 0.00 |  | Colon | 0.00 | 0.00 | 0.00 | 0.00 | 0.00 |
| Gonads | 0.00 | 0.00 | 0.00 | 0.00 | 0.00 |  | Gonads | 0.00 | 0.00 | 0.00 | 0.00 | 0.00 |
| Activemarrow | 0.01 | 0.00 | 0.00 | 0.00 | 0.01 |  | Activemarrow | 0.00 | 0.00 | 0.00 | 0.00 | 0.01 |

**Supplemental table 11: Product information**

| **Software used** | **Manufacturer, city, country** |
| --- | --- |
| National Cancer Institute dosimetry system for Radiography and Fluoroscopy (NCIRF) software (NIH Ref. No. E-127-2023) | National Cancer Institute, Bethesda, MD |
| National Cancer Institute dosimetry system for Computed Tomography (NCICT) software (NIH Ref. No. E-082-2016-0) | National Cancer Institute, Bethesda, MD |
| DICOMInspector software | Luxembourg Institute of Science and Technology (LIST), Esch-sur-Alzette, Luxembourg |
| **Machines used in Spain (Computed tomography)** |  |
| Philips Mx8000 CT | Philips Medical Systems (Cleveland) Inc., Cleveland, OH |
| Philips Brilliance 64 | Philips Medical Systems Nederland B.V., Best, Netherlands |
| Philips Ingenuity CT | Philips Healthcare Co., Ltd., Suzhou, China |
| Philips iCT256 | Philips Medical Systems Nederland B.V., Best, Netherlands |
| Philips Brilliance Big Bore | Philips Medical Systems (Cleveland) Inc., Cleveland, OH |
| Siemens Somatom Definition AS+ | Siemens Healthineers, Erlangen, Germany |
| **Machines used in Spain (Chest radiography)** |  |
| Philips DigitalDiagnost C90 | Philips Medical Systems DMC GmbH, Hamburg, Germany |
| Philips Medical Systems PCR AC3 | Philips Medical Systems Nederland B.V., Best, Netherlands |
| Philips Medical Systems, PCR Eleva | Philips Medical Systems DMC GmbH, Hamburg, Germany |
| Siemens FD-X (Flat Detector X-ray) | Siemens Healthineers, Erlangen, Germany |
| Agfa CR 30-X | Agfa HealthCare, Peissenberg, Germany |
| Agfa DX-M | Agfa-Gevaert N.V., Mortsel, Belgium |
| DRGEM GXR System | DRGEM Corporation, Gyeonggi-do, South Korea |
| GE HealthCare "Feitian Platform" | GE HealthCare, Beijing, China |
| **Machines used in Mozambique (Chest radiography)** |  |
| Philips PrimaryDiagnost AR | Philips Medical Systems DMC GmbH, Hamburg, Germany |
| Siemens Multix Fusion | Siemens Healthineers, Erlangen, Germany |
